# A collisional model for AFM manipulation of rigid nanoparticles

**DOI:** 10.3762/bjnano.1.19

**Published:** 2010-12-22

**Authors:** Enrico Gnecco

**Affiliations:** 1 Department of Physics, University of Basel, Klingelbergstrasse 82, 4056 Basel, Switzerland; present address: IMDEA Nanociencia, Campus Universitario de Cantoblanco, Avda. Fco Tomás y Valiente 7, 28049 Madrid, Spain

**Keywords:** atomic force microscopy, nanomanipulation, nanoparticles

## Abstract

The trajectories of differently shaped nanoparticles manipulated by atomic force microscopy are related to the scan path of the probing tip. The direction of motion of the nanoparticles is essentially fixed by the distance *b* between consecutive scan lines. Well-defined formulas are obtained in the case of rigid nanospheres and nanowires. Numeric results are provided for symmetric nanostars. As a result, orienting the fast scan direction perpendicular to the desired direction of motion and reducing *b* well below the linear size of the particles turns out to be an efficient way to control the nanomanipulation process.

## Introduction

Quite soon after its invention, it became clear that atomic force microscopy (AFM) could be used not only for maging but also for manipulating nano-objects [1–2]. This possibility has produced spectacular results and last, but not least, it has allowed the controlled manipulation of metal clusters on insulating surfaces [3] and even single atoms on semiconductors [4]. However, AFM manipulation tends to be time-consuming. A major issue is that nanoparticles are usually moved individually so that the AFM tip has to be properly positioned with respect to the particle every time. The tip is either placed on the side or on the top of the particle. Then the tip–particle interaction is increased (by varying the tip–particle distance or the amplitude of the tip oscillations) until the particle is detached from the substrate and moved in a direction which is determined by several factors such as the scan pattern, the surface structure and the geometry of both tip and particle. Predicting the direction of motion of nanoparticles is very important, especially if it is desired to manipulate several particles at the same time. Here, we show that this is possible in simple cases of practical interest. Speciﬁcally, we assume that the AFM is operated in tapping mode (although some conclusions may be extended to contact mode), the particles are rigid and the frictional forces between particles and substrate can be neglected when the particles collide with the tip, but they are high enough to stop the particles immediately once contact with the tip is lost. The concentration of nanoparticles on the substrate is also supposed to be low enough to prevent multiple collisions in the manipulation.

After a brief review of previous results on the manipulation of rigid nanorods, including nanospheres and thin nanowires as limit cases, we discuss symmetric nanostars as a prototype of more complex shaped particles. We show that in any case the angle of motion of the nanoparticles is precisely related to the distance *b* between consecutive scan lines. When the parameter *b* is sufficiently small, the particle tends to move perpendicularly to the scan direction. The exact relation between the angle of motion θ and the parameter *b* depends on the particle shape and can be, in principle, determined analytically. Curiously, this has a certain analogy to the scattering of sub­atomic particles, whose angle of deﬂection θ depends on the form of the scattering ﬁeld and on the impact parameter *b* (i.e., the distance at which the particle would pass the center of the ﬁeld in the absence of any interaction) [5].

## Results

### The model

**Figure 1:**
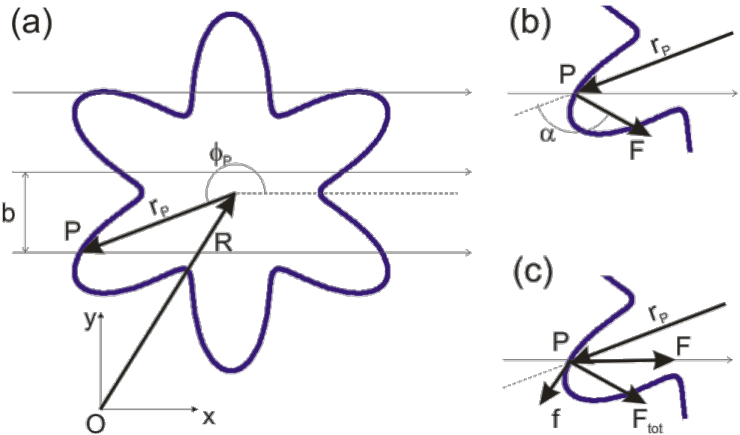
(a) A sharp nanotip follows a raster scan pattern with consecutive scan lines separated by a distance *b*. The tip collides with a nanoparticle (here represented by a star-shaped island) at the location *P*. (b) In tapping mode the tip oscillates in the direction *z* perpendicular to the plane of the ﬁgure and applies an impulsive force **F** perpendicular to the island proﬁle. (c) In contact mode the force **F** is directed along the *x* axis and the total force acting on the particle will be oriented as in tapping mode only if the static friction **f** can balance the component of **F** along the island proﬁle.

We ﬁrst consider a planar island whose proﬁle is described by the function *r* = *r*(φ) in polar coor­dinates or, equivalently, by a multi-value function *y* = *y*(*x*) in cartesian coordinates. Assuming that the tip follows a raster scan pattern, the *y* coordinate of the tip varies as *Y*_0_ = *Nb*, where *N* is the number of the scan line and *b* is the distance between consecutive scan lines (Figure 1a).

The island has a mass *M* and a moment of inertia *I* with respect to the normal axis *z* through its center of mass (COM). Here, we assume that the linear size of the island is much larger than the tip radius, so that the force **F** applied by the tip is concentrated at the point of contact *P*. We also assume that the island cannot be deformed or broken during the manipulation. In such a case, the position **R** ≡ (*X*,*Y* ) of the COM and the angle of rotation Φ of the island about the normal axis *z* evolve according to the equations of motion of a rigid body:

[1]
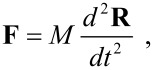


and

[2]
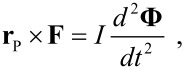


where **r**_P_ deﬁnes the position of the point of contact *P* with respect to the COM.

The direction of the force **F** depends on the operating mode of the AFM. In tapping mode the tip oscillates in the *z* direction with a frequency in the order of 100 kHz with an amplitude of some tens of nm. This corresponds to an average speed of some mm/s, which is well above typical scan velocities in AFM (normally in the order of 1 µm/s). Thus, the tip hits the particle almost vertically and the vector **F** is oriented perpendicularly to the island proﬁle, i.e., at an angle α = β + 90^◦^ with respect to the *x* axis, where


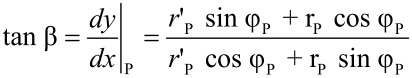


and *r*' is the ﬁrst derivative of *r*(φ) with respect to φ (Figure 1b). In contact mode the tip hits the particle along the *x* direction and the force **F** can be oriented as in tapping mode only if the static friction force **f** between tip and particle is high enough to prevent sliding along the island proﬁle (Figure 1c).

Assuming that friction between island and substrate is also high enough to prevent any slippage of the island after a collision with the tip, Equation 1 and Equation 2 can be averaged over the short collision time Δ*t* (in the order of 1/ *f* , with *f* ~ 10^5^ Hz being the oscillation frequency of the tip). This leads to the equations

[3]
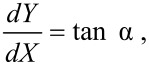


for the translation of the island and

[4]
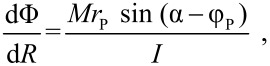


for the rotation, which can be ﬁnally integrated over the total time of interaction between tip and particle (along the given scan line). If the nanoparticle is not ﬂat, it is easy to see that the previous analysis is still applicable provided that the particle does not roll and that its shape is not cylindrical.

### Translation and wobbling of nanorods

The manipulation of a rigid nanorod formed by a cylinder (with length *L*) and two hemispherical caps (with radius *a*) is particularly instructive. Here, any possible rolling can be ignored and we can distinguish between two types of collision: (a) The tip touches the cylindrical core of the nanorod (“core” collision). (b) The tip touches one of the two hemispherical ends of the rod (“cap” collision). In case (a) the equations of motion of the nanorod can be written in the form [6]


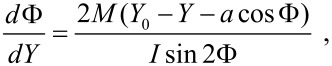


and


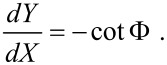


In the case (b):

[5]
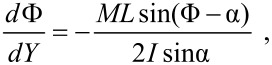


and

[6]
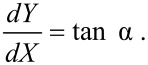


In general, both core and cap collisions occur along each scan line and only numerical solutions are possible. However, a complete solution can be found in two important cases: The manipulation of a nanosphere of radius *a* (*L* = 0) and that of a thin nanowire of length *L* (*a* = 0), where only cap collisions or core collisions, respectively, occur. In the case of a nanosphere, Equation 5 and Equation 6 can be integrated leading to the following result [7]. The direction of motion of the sphere forms an angle θ with respect to the *x* axis (fast scan direction) given by

[7]
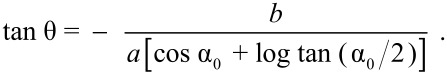


The quantity α_0_ is the impact angle between tip and sphere (with the exception of the very ﬁrst collision) and is given by


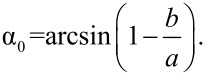


In the case of a nanowire, the *average* direction of motion is well-deﬁned and is given by the sim­ple formula [6]

[8]
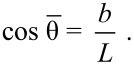


The wire oscillates perpendicularly to this direction:


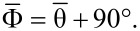


**Figure 2:**
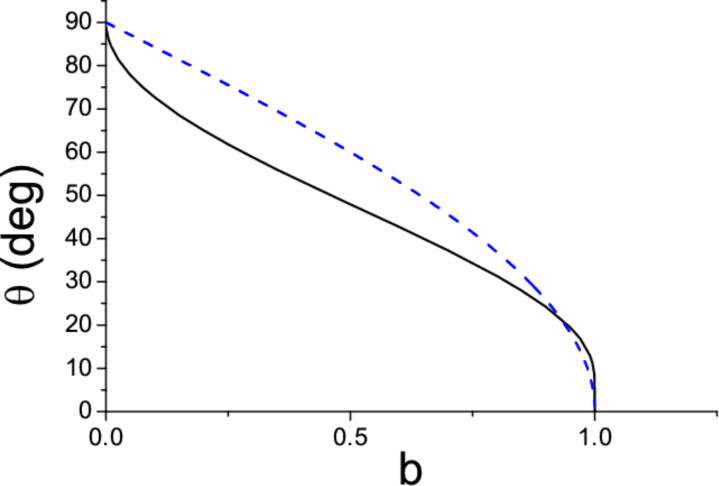
Angle of motion θ of a nanosphere (solid curve) and a nanowire (dashed curve) as a function of the distance *b* between consecutive scan lines. The parameter *b* is expressed in units of the sphere radius *a* and wire length *L* respectively.

Thus, Equation 7 and Equation 8 show that the directions of motion of nanospheres and nanowires manipulated by AFM in tapping mode are completely determined by the distance *b* between consecutive scan lines or, equivalently, by the density of scan lines 1/*b*. The functions of Equation 7 and Equation 8 are plotted in Figure 2. In both cases θ(*b*) decreases with increasing *b* until the particle is lost when *b* > *a* or *b* > *L*. Furthermore, the angle θ → 90^◦^ when *b* → 0. Numerical simulations show that similar conclusions are also valid for arbitrarily thick nanorods [6], although simple analytic expressions cannot, in general, be derived.

### Star shaped islands: Rotational effects

As a next step we extend our analysis to more complex shapes. We consider star-shaped islands, whose proﬁle is described by the function


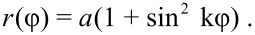


**Figure 3:**
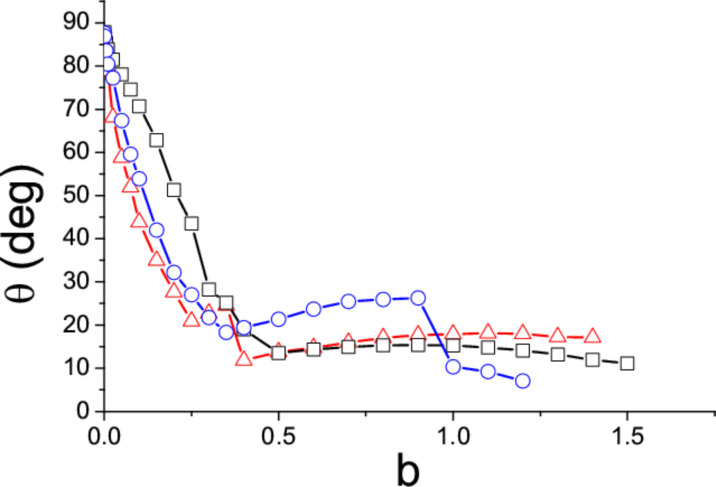
Angle of motion θ of 2*k*-branched symmetric islands as a function of the distance *b* be­tween consecutive scan lines (in units of the length parameter *a* in the text). *k* = 2 (squares), *k* = 3 (circles) and *k* = 4 (triangles).

**Figure 4:**
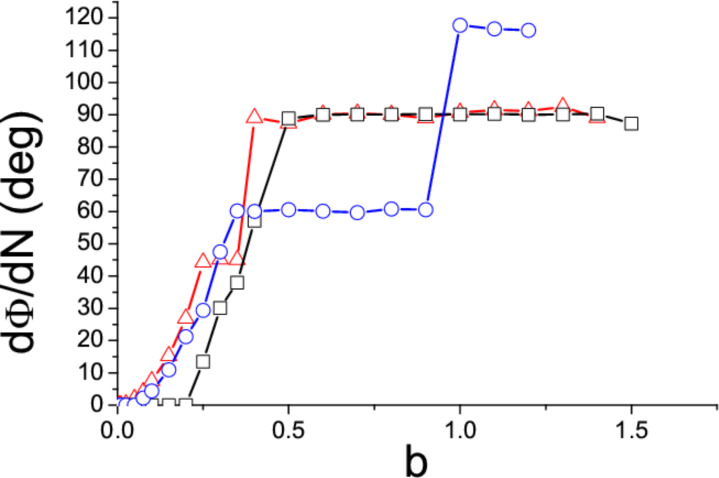
Angular velocity of the islands as a function of *b*. *k* = 2 (squares), *k* = 3 (circles) and *k* = 4 (triangles).

The number of branches in the island is denoted by 2*k*. For instance, *k* = 3 in Figure 1a. It is inter­esting to observe that both the moment of inertia *I* and the area *A* of the island are independent of *k*:


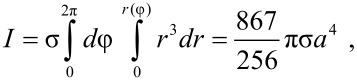



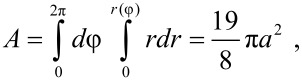


where σ = *M*/*A* is the area density of the island. The ratio *M*/*I* which appears in Equation 4 is thus equal to (867/608)*a*^2^ = 1.426*a*^2^. The equations of motion (Equation 3 and Equation 4) have been solved for *k* = 2,3,4 and increasing values of *b* until the island starts moving in the negative *y* direction and is lost. In Figure 3 the angle of motion θ = arctan(*dY*/*dX*) is plotted as a function of the parameter *b*. The initial coordinate *Y*_0_ of the tip along the slow scan direction was randomly chosen, with hardly any inﬂuence on the ﬁnal results, except in the threshold region where the islands can be lost (and no points can be plotted). In all cases the direction of motion θ initially decreases with increasing *b* and, again θ → 90^◦^ when *b* → 0. However, the trend of the function θ(*b*) suddenly changes when *b* reaches a certain value (*b* = 0.5, 0.35 or 0.25 when *k* = 2, 3 or 4). In order to understand what happens at these points, we have also plotted the angular velocity of the particles, *d*Φ/*dN*, as a function of *b* (Figure 4). The critical values of the parameter *b* correspond to the onset of rotations of 180^◦^/*k* angles per scan line. Beyond these critical values the angular velocity remains almost constant and the function θ(*b*) slightly increases (Figure 3). When *k* = 3 and 4 two further critical values of *b* are found (*b* = 0.9 and 0.35 respectively), corresponding to rotations of 2 × 180^◦^/*k* angles per scan line. On the other hand, when *b* is small enough, the angular velocity *d*Φ/*dN* becomes negligible: Rather than rotating, the islands simply ‘wobble’ like the nanorods.

## Discussion

The predictions of the collisional model have been experimentally veriﬁed with gold nanospheres and nanorods manipulated on silicon oxide under ambient conditions by tapping AFM [6–7]. Furthermore, we have also observed that, at least in the case of the nanospheres, the model goes beyond the restrictive hypothesis that the particles are immediately stopped after being released by the tip. This has been shown by numerical simulations, where a ‘mean free path’ *d* of the nanoparticles was introduced. If the friction force between particle and substrate decreases, and consequently the distance *d* increases, then the pathway of the nanoparticle ﬂuctuates more and more, but the form of the function θ(*b*) remains essentially unchanged [8]. Another important point is the following. In many commercial AFMs, the tip follows a zigzag scan path rather than a raster scan path. This leads to signiﬁcant variations in the impact angles between the tip and particles and to a dependence of the direction of motion on the initial position of the particles along the fast scan direction *x* [7]. Nevertheless, at least in the case of nanospheres, one of the previous conclusions holds: The angle of motion θ → 90^◦^ when *b* → 0 (in the case of a zigzag scan pattern, *b* can be taken as the distance between the starting points of parallel scan lines). Altogether, these observations suggest a general strategy for manipulating relatively large nanoparticles, i.e., in the order of or larger than the tip radius. Provided that the density of scan lines is high enough, the direction of motion of the particles can be tuned by orienting the fast scan direction *x* of the AFM *perpendicularly* to the desired direction of motion. This is much easier and more reliable than aligning the tip and moving it towards the COM of each nanoparticle, as is usually done. The rotational effects predicted by the collisional model have not yet been tested experimentally. A good benchmark would be the ﬂower-shaped Sb islands ﬁrst manipulated by Ritter et al. on HOPG and MoS_2_ [9]. Possible discrepancies between theory and experiment concerning the direction of motion and angular speed of the islands could be related to the friction forces between island and substrate and even used to estimate these forces in further developments of the collisional model. Since Sb islands can be manipulated and the corresponding friction forces can be measured also in contact mode [10], the applicability of the model could also be tested under these different impact conditions. Controlling the direction of motion of arbitrarily shaped nanoparticles is important for the guided formation of nanostructures. An interesting analogy is found with AFM nanolithography. In a recent paper we have shown that the patterning of amorphous polymers can be ‘tuned’ by varying the scan path of an AFM tip which scratches the polymer surface while scanning [11]. Linear and ‘travelling’ circular ripples were formed using a raster or a circular scan path, respectively. In the same way, a desired conﬁguration of nanoparticles could be obtained by a proper choice of the scan pattern.

## Conclusion

In conclusion, we have shown that the direction of motion of nanoparticles can be controlled by AFM in a variety of signiﬁcant cases. The key parameter is simply the density of scan lines in the scan path of the probe tip. Orienting the fast scan direction perpendicularly (and not parallel) to the desired direction of motion is an efﬁcient way for manipulating the nanoparticles. With a proper choice of the scan pattern, it may be possible to reorganize an ensemble of randomly distributed nanoparticles in a well-deﬁned arrangement.
